# Discrimination of the Expression of Paralogous microRNA Precursors That Share the Same Major Mature Form

**DOI:** 10.1371/journal.pone.0090591

**Published:** 2014-03-03

**Authors:** Minghua Wang, Weiping Wang, Ping Zhang, Juanjuan Xiao, Jianguo Wang, Chaoqun Huang

**Affiliations:** 1 Department of Biochemical and Molecular Biology, Medical College, Soochow University, Suzhou, Jiangsu, China; 2 Department of Physiological Sciences, Oklahoma State University, Stillwater, Oklahoma, United States of America; Laboratoire de Biologie du Développement de Villefranche-sur-Mer, France

## Abstract

**Background:**

MicroRNAs (miRNAs) are a class of small non-coding RNAs generated from endogenous transcripts that form hairpin structures. The hairpin precursor is processed into two mature miRNAs that form major/minor duplexes. Mature miRNAs regulate gene expression by cleaving mRNA or repressing protein translation. Numerous miRNAs have been discovered via deep sequencing. Many miRNAs are produced from multiple genome sites. These miRNAs are grouped into paralogous families of miRNAs that generate the same major mature form within organisms. Currently, no method of distinguishing the expression of these miRNAs is available.

**Results:**

In the present study, strategies were developed to discriminate and quantify the expression of paralogous miRNA precursors. First, paralogous miRNA precursors that were differentially expressed in tissues were identified through analysis of the coexpression scores of their major and minor forms based on deep sequencing data. Then the precursors were identified by monitoring the expression of their host gene or minor form using real-time PCR. Finally, precursors were identified by assessing the expression of clusters of miRNA members. These approaches were used to distinguish miR-128-1 and miR-128-2 as well as miR-194-1 and miR-194-2. The mechanism of transcription related to the differential expression of miR-194-1 and miR-194-2 was also investigated.

**Conclusion:**

This is the first report to distinguish paralogous miRNA copies by analyzing the expression of major-minor pairs, the host gene, and miRNA clusters. Discriminating paralogous precursors can provide useful information for investigating the mechanisms that regulate miRNA gene expression under different physiological and pathological conditions.

## Introduction

MicroRNAs (miRNAs) are a class of conserved small molecular non-coding RNAs of about 22 nucleotides in length. They regulate gene expression through mRNA cleavage and translational repression by binding to the 3′ untranslated region of the target mRNA. miRNAs also play important roles in cell development, differentiation, proliferation, and oncogenesis.[1]–[4] miRNA genes are transcribed to generate pri-miRNAs, which are intergenic and intronic primary RNA transcripts. Pri-miRNAs are processed into ≈70 nt hairpin precursors (pre-miRNAs) by Drosh.[5], [6] The miRNA precursors are then exported into the cytoplasm and recognized by Dicer. They are eventually processed into mature small miRNAs.[6], [7] Many miRNAs have been discovered through deep sequencing.[8] Many miRNAs are produced from multiple sites in genome. Closely related miRNAs are grouped into paralogous families that have related functions.[9] Furthermore, some paralogous miRNA genes produce identical nucleotide sequences of major mature forms within organisms. If the miRNA reads map to more than one precursor sequence, it can be difficult to distribute the sequence counts to the correct one. After being processed by Dicer, the miRNA precursor is divided into two different mature miRNAs, a stable major form and an unstable minor form.[10] These two mature miRNAs form a major/minor duplex. Although the minor form degrades rapidly, significant amounts can be detected through deep sequencing. The major form is usually well conserved but the minor form is not. The sequence of the minor form may differ across paralogous precursors. In this way, these paralogous miRNA precursors can be distinguished by detecting the coexpression of major/minor pairs.

Some miRNAs are located in the same transcription unit as other RNAs that encode proteins or non-coding RNAs. The miRNA host gene encodes this kind of transcript, which is usually expressed in parallel with host genes. The expression patterns of miRNA host genes are monitored to demonstrate physiologic and pathologic regulation of miRNA expression.[11] More than 40% of miRNAs are located in the introns or exons of other RNA transcripts in a sense or antisense direction.[11] miRNA is usually regulated along with the host gene if they are both in the sense orientation.[12] Paralogous miRNA precursors that share the same mature form may be produced from different host transcripts. In this way, the expression of paralogous precursors may be determined by monitoring the host gene.

A set of two or more miRNAs forms a cluster, which is transcribed in the sense orientation from physically adjacent miRNA genes. miRNA clusters perform important functions by regulating various cellular processes. For example, the most frequently studied miR-17-92 cluster is involved in the development of the heart, lungs, and immune system and in the formation of tumors.[13]–[15] Clustered miRNAs are usually derived from a common primary transcript and coexpressed.[16] Paralogous miRNA precursors that share the same mature form may also be members of different miRNA clusters. In this way, the expression of paralogous precursors may be distinguished by assessing the expression of cluster miRNAs.

Some paralogous miRNA genes were processed to the same major mature form. In this way, it is impossible to distinguish these paralogous miRNA precursors by analyzing the expression of their major forms. In the present study, approaches to the discrimination of the expression of these miRNA precursors were developed based on miRNA deep sequencing data and real time PCR analysis. In addition, the transcription mechanisms related to the differential expression of miRNA precursor family were investigated.

## Results

### Tissue-specific and -selective expression of paralogous miRNAs

The miRBase database release 19 contains 855 precursor and 1281 mature mouse miRNAs. Of these, 55 paralogous miRNA families were found. These consist of 138 precursors. Tissue-specific expression of these 55 mature miRNAs, which were processed from 138 precursors, was analyzed. PaGeFinder analysis was performed on 55 mature miRNAs based on the reads generated by RNA deep sequencing. The results, shown in **Table 1**, showed that 19 of these 55 mature miRNAs were expressed in a tissue-specific or -selective manner.

**Table 1:** Identification of miRNAs tissue specifically or selectively expressed.

Precursor	Mature	Tissue expression
mmu-miR-124-1,2,3	mmu-miR-124-3p	brain
mmu-miR-128-1,2	mmu-miR-128-3p	thymus, brain
mmu-miR-129-1,2	mmu-miR-129-5p	brain
mmu-miR-133a-1,2	mmu-miR-133a-3p	skeletal muscle, heart, skin
mmu-miR-135a-1,2	mmu-miR-135a-5p	brain, salivary gland
mmu-miR-138-1,2	mmu-miR-138-5p	brain
mmu-miR-181a-1,2	mmu-miR-181a-5p	thymus
mmu-miR-181b-1,2	mmu-miR-181b-5p	thymus
mmu-miR-194-1,2	mmu-miR-194-5p	liver, kidney
mmu-miR-196a-1,2	mmu-miR-196a-5p	kidney
mmu-miR-199a-1,2	mmu-miR-199a-5p	skin
mmu-miR-1a-1,2	mmu-miR-1a-3p	skeletal muscle, heart
mmu-miR-218-1,2	mmu-miR-218-5p	brain
mmu-miR-219-1,2	mmu-miR-219-5p	brain
mmu-miR-344-1,2	mmu-miR-344-3p	brain
mmu-miR-344d-1,2,3	mmu-miR-344d-3p	brain
mmu-miR-465b-1,2	mmu-miR-465b-3p	testes
mmu-miR-465c-1,2	mmu-miR-465c-3p	testes
mmu-miR-9-1,2,3	mmu-miR-9-5p	brain

PaGeFinder analysis was performed using the normalized reads of miRNA major form. The search criteria were set to SPM>0.9 for the identification of tissue-specific miRNAs or at SPM>0.5 and CTM>0.9 for the identification of tissue-selective miRNAs.

### Associations between major and minor mature forms and among clustered miRNAs

We now determined whether major and minor mature forms from the same precursor were co-expressed in 15 mouse tissues. Here miRNA precursors with total reads of major form more than 50 in 15 mouse tissues were selected. The Pearson correlation R-values and P-values in 419 major/minor pairs were calculated. As shown in **Table S5**, 304 significantly correlated pairs were found (p<0.01). The expression of 73 percent (304 to 419) of major and minor pairs was positively correlated. We also investigated whether clustered microRNAs are co-expressed. 52 miRNA clusters with inter-miRNA distance<5000 bp (base pair) were selected. Pearson correlation R-values and P-values in 865 miRNA pairs in these clusters were computed. As shown in **Table S6**, 693 clustered miRNA pairs are significantly correlated (p<0.01) in our datasets. The expression of 80 percent (693 to 865) of clustered miRNA pairs was positively correlated.

### Expression profile of paralogous miRNA precursor and major mature form

**Figure 1:**
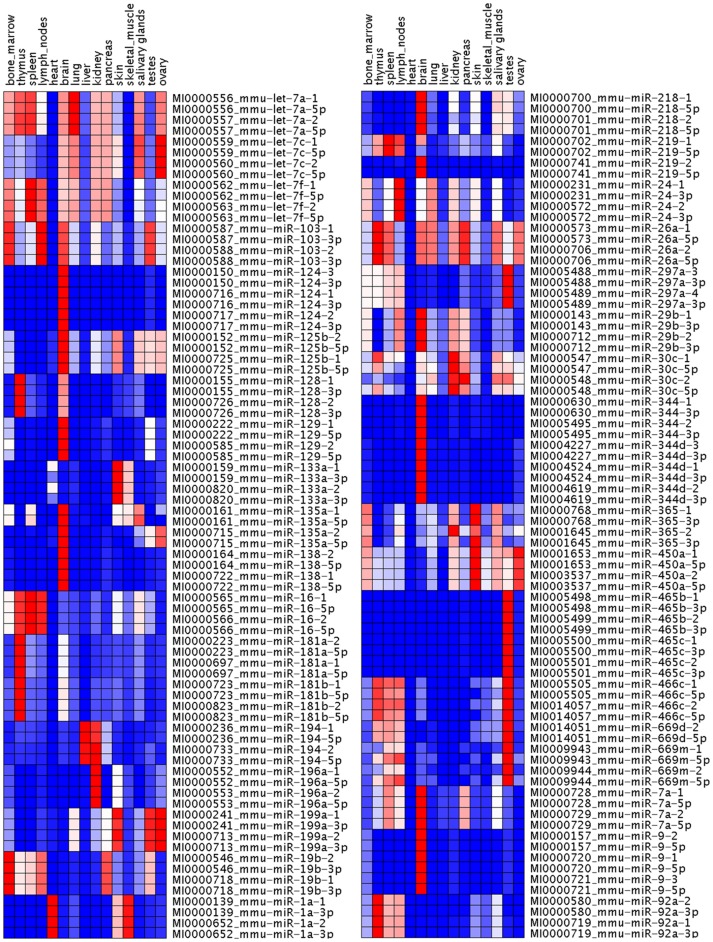
Expression of miRNA precursor and its major mature form in 15 mouse tissues. A heat map was constructed using GenePattern software based on the normalized miRNA reads. Heat maps showed precursors and major forms to have similar patterns of expression.

A relative expression analysis of precursors and their corresponding major forms was performed across different mouse tissues. All reads that mapped to particular precursor were used to analyze the expression of that precursor. Reads with the same start position as a particular mature miRNA were also used to analyze the expression of mature miRNA, and the greatest number of reads that mapped to a particular precursor was considered the expression of that major form (**Table S2**). Each miRNA count from each mouse tissue was normalized to the total number of reads for that tissue. The normalized reads were used to build a heat map using GenePattern software. The results shown in **Figure 1** showed the hairpin precursor and the major product to almost always be extremely well matched in the expression profiles of most miRNAs. However, the expression of some precursors including miR-219-1 and miR-219-2, miR-30c-1and miR-30c-2, miR-365-1 and miR-365-2 could be distinguished from the heatmap. miR-219-2 was preferentially expressed in brain, but miR-219-1 was preferentially expressed in spleen and lymph nodes. miR-365-2 was preferentially expressed in kidney, but miR-365-1 was not.

### Discrimination of paralogous precursors using the coexpression of major and minor forms

**Figure 2:**
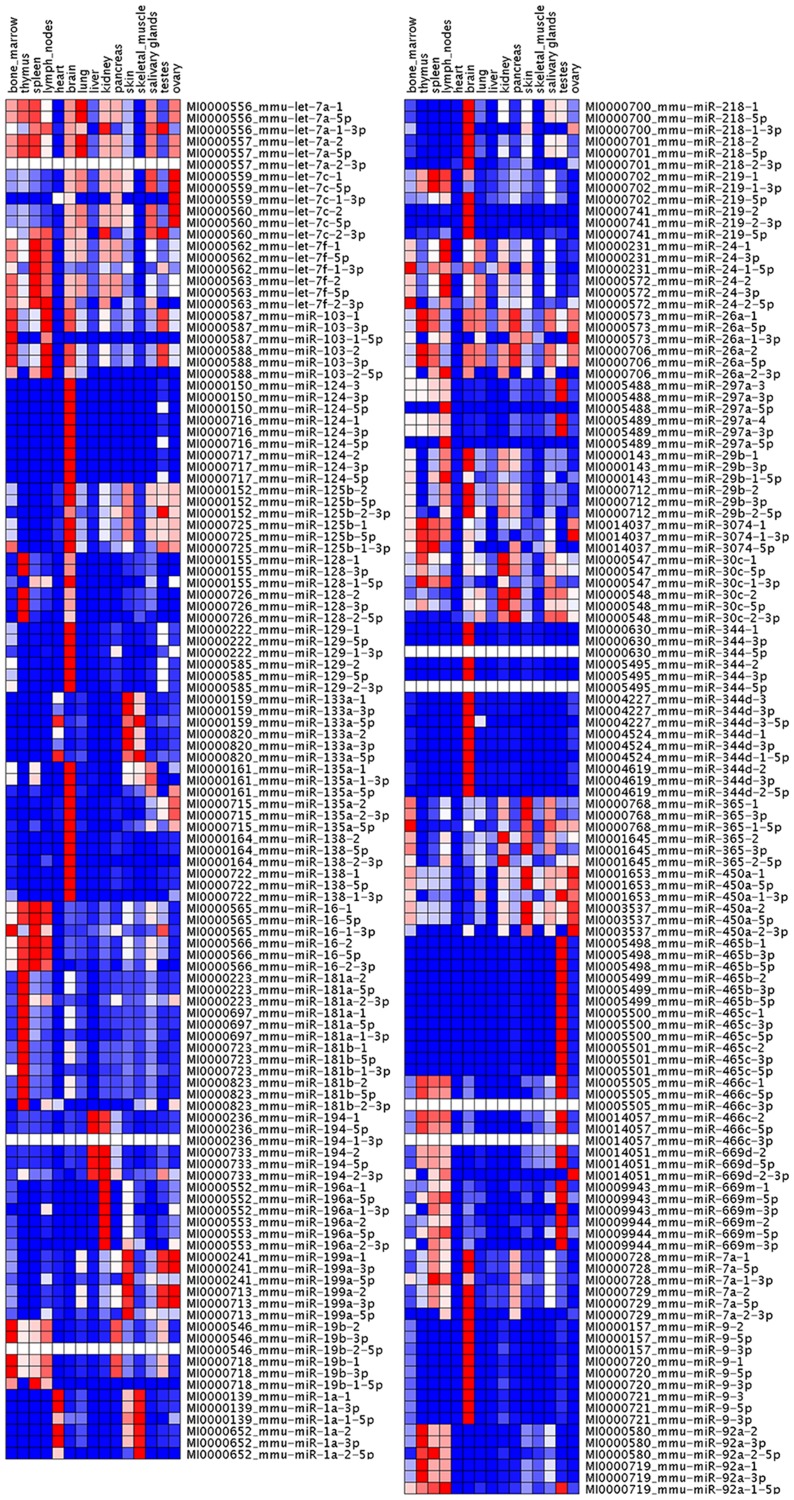
Expression of the miRNA precursor and its major and minor mature forms in 15 mouse tissues. Heat maps were constructed using GenePattern software based on the normalized miRNA reads. This heat map shows precursors and minor forms to have different patterns of expression in many paralogous families.

To distinguish the expression of paralogous precursors that share the same major mature form, we determined the occurrence of major/minor pairs in 15 normal mouse tissues. The biological hypothesis tested here involved analysis of the coexpression of two forms of miRNAs in the same tissue. Coexpression was considered indicative of precursors with high probability of being processed into the corresponding major/minor pair. The miRNA precursors were first excluded if the expression of major and minor forms in this precursor family were not significantly associated by Pearson's correlation analyses. The top 10% of the scored major/minor pairs were then selected (**Table S3**). Next the minor forms with *P* values below 0.05 in the Fisher's Exact Test were used as standards to further filter the major/minor pairs. The major/minor pairs that were finally selected for evaluation of the tissue-differential expression of miRNA precursors are listed in **Table 2**. The normalized reads of mature and precursor miRNAs were also used to build a heatmap using GenePattern software. The results shown in **Figure 2** indicate that different minor forms processed from precursors sharing the same major form had different patterns of expression pattern.

**Table 2:** Tissue coexpression scores of major/minor pairs.

Precursor	Major/minor	BM	Thy	Spl	Lym	Bra	Lun	Liv	Kid	Pan	Ski	Sal	Tes	Ova
let-7a-1	let-7a-5p/let-7a-1-3p			1960		294	487		455			449		
let-7a-2	let-7a-5p/let-7a-2-3p			0		0	0		0			0		
miR-103-1	miR-103-3p/miR-103-1-5p				562									
miR-103-2	miR-103-3p/miR-103-2-5p				3475									
miR-125b-1	miR-125b-5p/miR-125b-1-3p	422									233			
miR-125b-2	miR-125b-5p/miR-125b-2-3p	0.1									1934			
miR-128-1	miR-128-3p/miR-128-1-5p		0											
miR-128-2	miR-128-3p/miR-128-2-5p		64394											
miR-129-1	miR-129-5p/miR-129-1-3p					34496								
miR-129-2	miR-129-5p/miR-129-2-3p					108352								
miR-135a-1	miR-135a-5p/miR-135a-1-3p					95								0
miR-135a-2	miR-135a-5p/miR-135a-2-3p					4562								658
miR-16-1	miR-16-5p/miR-16-1-3p		0.7		93									
miR-16-2	miR-16-5p/miR-16-2-3p		337		1245									
miR-181a-1	miR-181a-5p/miR-181a-1-3p		1002528											
miR-181a-2	miR-181a-5p/miR-181a-2-3p		69507											
miR-194-1	miR-194-5p/miR-194-1-3p							0	0					
miR-194-2	miR-194-5p/miR-194-2-3p							783	987					
miR-1a-1	miR-1a-3p/miR-1a-1-5p										3092			
miR-1a-2	miR-1a-3p/miR-1a-2-5p										7.4			
miR-218-1	miR-218-5p/miR-218-1-3p					221								
miR-218-2	miR-218-5p/miR-218-2-3p					1425								
miR-219-1	miR-219-5p/miR-219-1-3p				222	0.3								
miR-219-2	miR-219-5p/miR-219-2-3p				0.0	42979								
miR-24-1	miR-24-3p/miR-24-1-5p						0.6							
miR-24-2	miR-24-3p/miR-24-2-5p						232							
miR-29b-1	miR-29b-3p/miR-29b-1-5p				1629	0.9			0.3					
miR-29b-2	miR-29b-3p/miR-29b-2-5p				177	863			172					
miR-7a-1	miR-7a-5p/miR-7a-1-3p			1879										
miR-7a-2	miR-7a-5p/miR-7a-2-3p			0										
miR-92a-1	miR-92a-3p/miR-92a-1-5p		26241	32193	16425									
miR-92a-2	miR-92a-3p/miR-92a-2-5p		9261	15280	34050									
miR-365-1	miR-365-3p/miR-365-1-5p	315							0.1					
miR-365-2	miR-365-3p/miR-365-2-5p	95							154					

Selection was based on the score ranked top 10% and Fisher exact test between minor forms with *P* values below 0.05 in particular tissues. If the minor form did not have any reads, then the coexpression score of its major/minor pair was considered to be zero.

BM: bone marrow, Thy: thymus, Spl: Spleen, Lym: lymph, Bra: brain, Lung: lung, Liv: Liver, Kid: kidneys, Pan: pancreas, Ski: skin, Sal: salivary glands, Tes: testes, Ova: ovary.

### Host gene expression of miRNA as a proxy for distinguishing paralogous precursor

**Figure 3:**
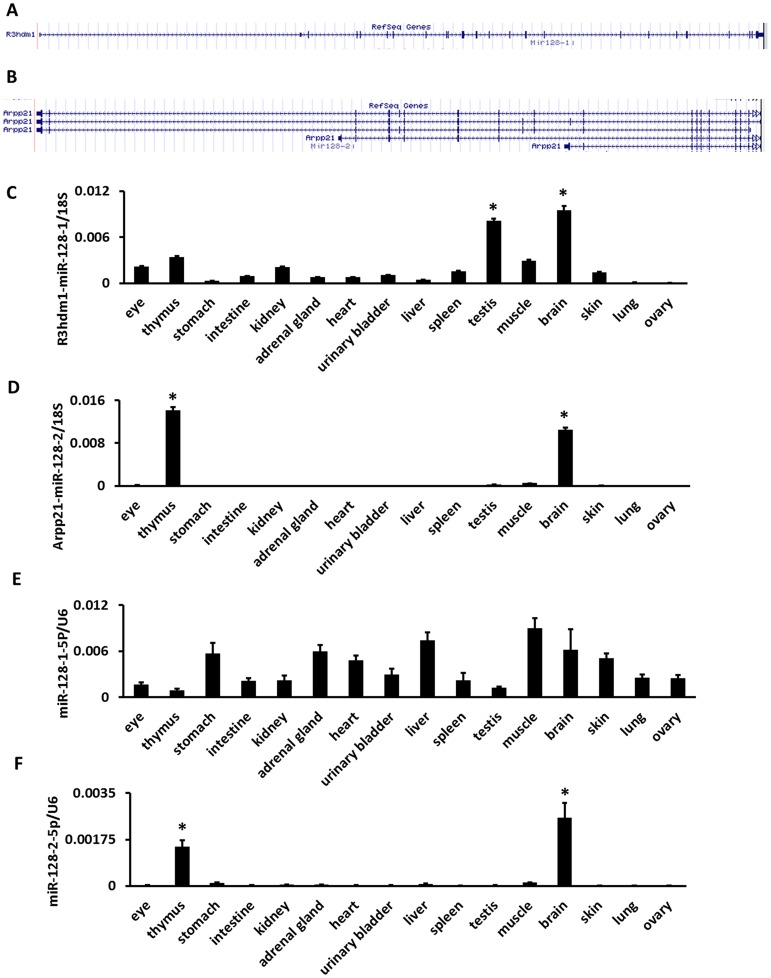
Locations and expressions of the host genes and minor forms of mouse miR-128-1, 2 in 16 mouse tissues. (a) Structure of R3hdm1 as miR-128-1 host gene. (b) Structure of Arpp21 as miR-128-2 host gene. (c) qRT-PCR analysis of R3hdm1 expression, the levels of expression were normalized to 18S rRNA. (d) qRT-PCR analysis of Arpp21 expression, the levels of expression were normalized to 18S rRNA. (e) qRT-PCR analysis of the expression of miR-128-1-5p and miR-128-2-5p, the levels of expression were normalized to U6 snRNA. * Preferential expressed tissues identified by ANOVA (*P*<0.05) and Turkey's HSD test.

The host gene of miR-128-1 and miR-128-2 here served as an example in a demonstration of the current method of distinguishing the expression of paralogous miRNA precursors. Both mmu-mir-128-1 and mmu-miR-128-2 are intronic miRNAs. mmu-mir-128-1 resides in the 16^th^ intron of the R3HDM1 gene on mouse chromosome 1qE3 (**Figure 3A**). mmu-miR-128-2 is embedded in the 17th intron of the ARPP21 gene on mouse chromosome 9qF3 (**Figure 3B**). mmu-miR-128-1 is processed into miR-128-3p and miR-128-1-5p. mmu-miR-128-2 is processed into miR-128-3p and miR-128-2-5p. miR-128-3p is the common major mature form of miR-128-1 and 2. The results shown in **Table 1** show that miR-128-3p was selectively expressed in the brain and thymus. As shown in **Table S3** and **Table 2**, the coexpression scores of miR-128-3p and miR-128-2-5p were 64394 and 8628 in the thymus and brain, respectively. The coexpression scores of miR-128-3p and miR-128-1-5p were 0 and 7241 in the thymus and brain, respectively. We further investigated the expression of R3HDM1 and ARPP21 gene in 16 mouse tissues using real time PCR. The results shown in **Figure 3C** and **Figure 3D** indicate that ARPP21 was selectively expressed in the thymus and brain, but R3HDM1 was not. The expression of minor mature forms miR-128-1-5p and miR-128-2-5p in 16 mouse tissues was assessed using real time PCR. We found miR-128-2-5p was selectively expressed in the thymus and brain (**Figure 3E** and **Figure 3F**). Next we determined whether miR-128-3p and its host genes, R3HDM1 and ARPP21, are co-expressed. Expression value of R3HDM1 and ARPP21 mRNA (Real-time PCR) and read counts of miR-128-3p (deep sequencing) in mouse tissues were obtained. Pearson correlation R-values and P-values of R3HDM1/miR-128-3p pair and ARPP21/miR-128-3p pair were computed. We found that the expression of ARPP21 and miR-128-3p was significantly correlated (R = 0.98, P<0.05) but the expression of R3HDM1 and miR-128-3p was not (R = 0.48, P = 0.134). These results suggest that miR-128-2 is the major contributor to the expression of miR-128-3p in the thymus, but miR-128-1 is not.

### Identification of paralogous miRNA precursors by the coexpression of cluster miRNAs

**Figure 4:**
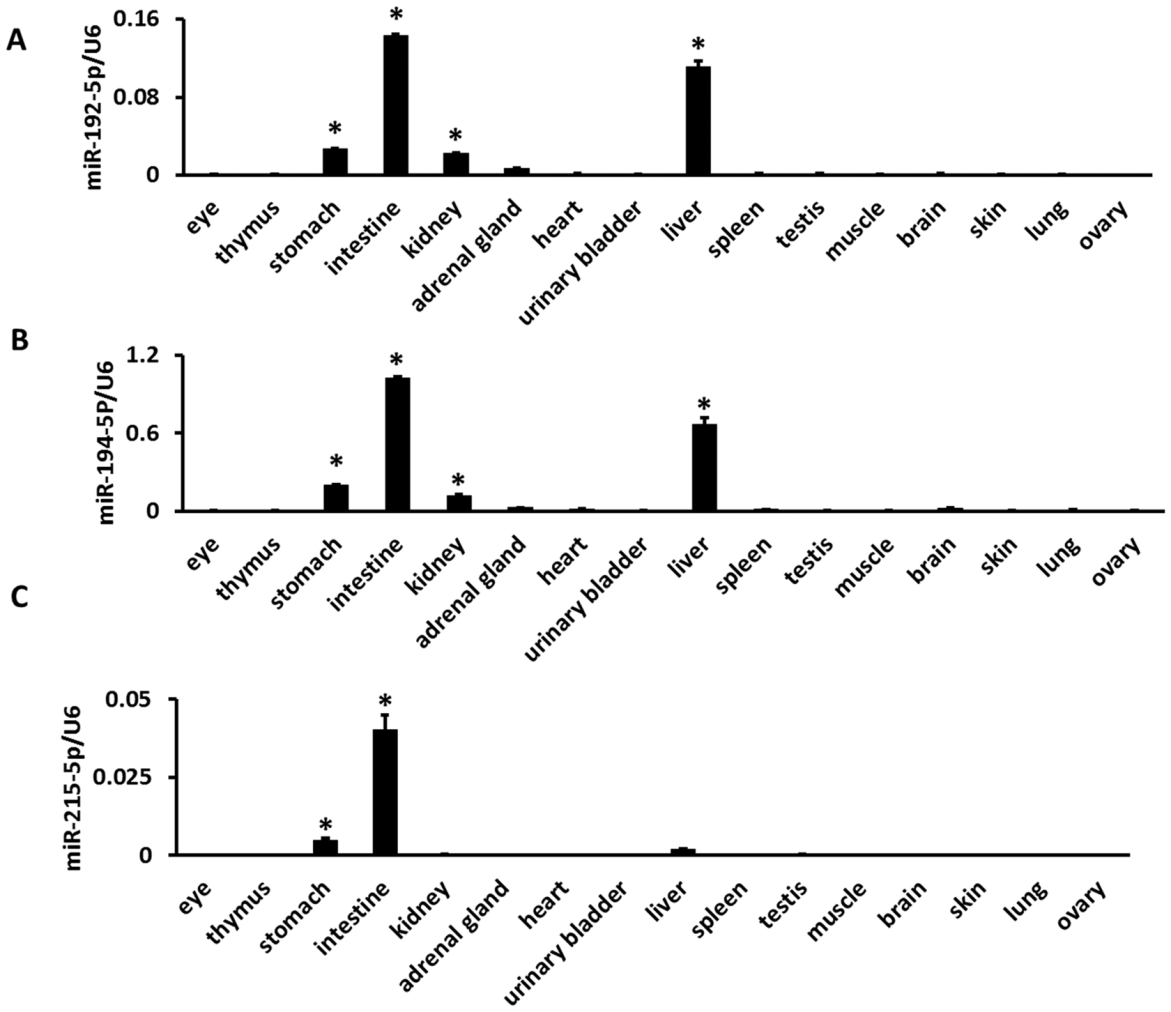
Expression of mature miRNAs in 16 mouse tissues. Expression analysis was performed using real-time RT-PCR. The expression levels were normalized to U6 snRNA. (a) miR-194-5p expression, (b) miR-192-5p expression, (c) miR-215-5p expression. * Preferential expressed tissues identified by ANOVA (*P*<0.05) and Turkey's HSD test

**Figure 5:**
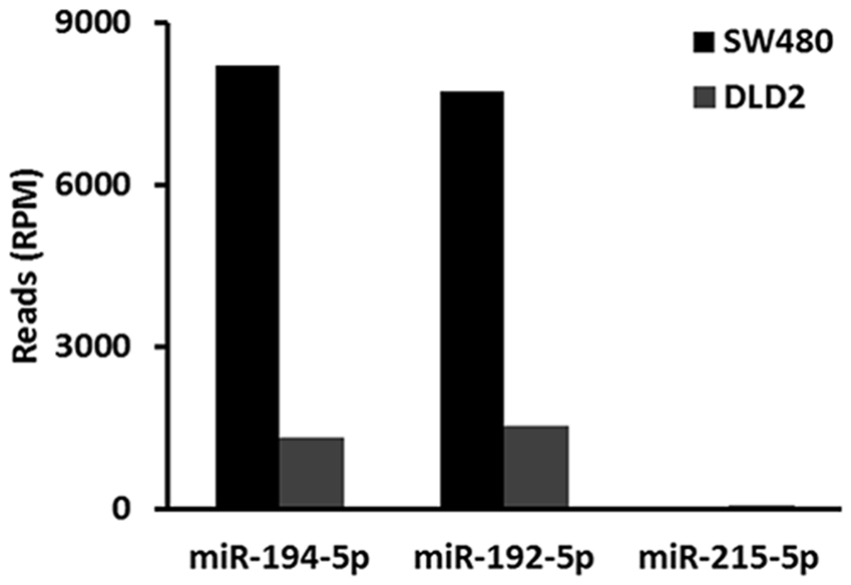
Expression of miR-194-5p, miR-192-5p, and miR-215-5p in human colon cell lines. The level of expression is given in sequence counts per million.

Here, the cluster containing miR-194-1/miR-215 and miR-194-2/miR-192 served as an example of how to distinguish the expression of paralogous miRNA genes. The mmu-miR-194-1 precursor is processed into miR-194-5p and miR-194-1-3p. mmu-miR-194-2 is processed into miR-194-5p and miR-194-2-3p. miR-194-5p is the common major mature form of miR-194-1 and miR-194-2. The results shown in **Table 1** above indicate that miR-194-5p was selectively expressed in the liver and kidneys. As shown in **Table 2**, the coexpression scores of miR-194-5p and miR-194-1-3p were 0 and 0 in the liver and kidneys, respectively. The coexpression scores of miR-194-5p and miR-194-2-3p were 783 and 987 in the liver and kidneys, respectively. miR-194-1 was found to cluster with miR-215 and miR-194-2 was found to cluster with miR-192. The coexpression of miR-194-5p and miR-192-5p and of miR-194-5p and miR-215-5p was calculated. As shown in **Table 3**, the coexpression scores of miR-194-5p and miR-215-5p were 0 in the liver and kidneys, respectively. The coexpression scores of miR-194-5p and miR-192-5p were 77,872 and 96,655 in the liver and kidneys, respectively. The expression of miR-194-5p, miR-192-5p, and miR-215-5p in 16 mouse tissues was assessed using real time PCR. miR-194-5p and miR-192-5p were selectively expressed in the gastrointestinal tract, liver, and kidneys (**Figure 4A, B**). However, miR-215 was found to be specifically expressed in the gastrointestinal tract but not in the liver or kidneys (**Figure 4C**). miR-215p also has much lower levels of expression in the gastrointestinal tract, liver, and kidney than miR-192-5p. Deep sequencing miRNA data are not available for the mouse gastrointestinal tract. However, such data are available for human cell lines DLD2 and SW480, isolated from the colon. The expression of miR-194-5p, miR-192-5p, and miR-215-5p were determined in DLD2 and SW480 cells. The results shown in **Table 4** and **Figure 5** indicate that both miR-194-5p and miR-192-5p are much more highly expressed in DLD2 and SW480 cells than miR-215-5p is. These results suggest that mmu-miR-194-2 was selectively expressed in the gastrointestinal tract, liver, and kidney and that mmu-miR-194-1 was specifically and minimally expressed in the gastrointestinal tract. Mmu-miR-194-2 is the major contributor to the expression of miR-194-5p in the gastrointestinal tract, liver, and kidneys.

**Table 3:** Tissue coexpression score of miR-194-5p/miR-215-5p and miR-194-5p/miR-192-5p.

Tissue	miR-194-5p/miR-215-5p	miR-194-5p/miR-192-5p
Bone marrow	56	4787
Thymus	13	596
Spleen	37	1867
Lymph nodes	53	3205
Heart	15	2820
Brain	21	995
Lung	0	1269
Liver	0	77872
Kidney	0	96655
Pancreas	0	0
Skin	0	1204
Skeletal muscle	73	3054
Salivary glands	0	908
Testes	0	39
Ovary	0	637

The coexpression score was zero for all miRNA with no reads.

**Table 4:** Reads per million of miR-194-5p, miR-192-5p, and miR-215-5p in human colon cell lines.

Cells	miR-194-5p (reads per million)	miR-192-5p (reads per million)	miR-215-5p (reads per million)
SW480	8208	7725	0
DLD2	1314	1527	47

### Mechanism of tissue differential expression of paralogous miRNA precursors

**Figure 6:**
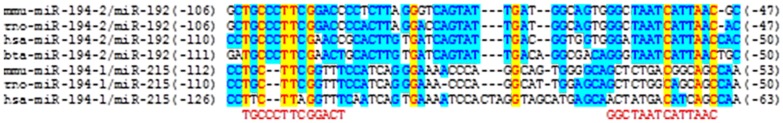
Potential HNF1 and HNF4 binding site in the promoters of pri-miR-194-1/miR-215 and miR-194-2/miR-192 genes in different species. Transcription binding sites were predicted using Motif-based sequence analysis tools.

Results showed miR-194-2 to be a major contributor to the expression of miR-194-5p in the liver and kidneys and showed the contribution of miR-194-1 to be negligible or nonexistent. Here the mechanism of differential expression of miR-194-1 and miR-194-2 was investigated. Based on the previously reported gene structure of pri-miR-194-2/miR-192, 2 kb promoter sequences of human, mouse, rat, and bovine pri-miR-194-2/miR-192 genes were downloaded.[17] In addition, by searching NCBI and UCSC genome database, cDNA AK008306 was found to be part of the transcript of pri-miR-194-1/miR-215. Based on the transcription start site of cDNA AK008306, 2 kb promoter sequences of mouse pri-miR-194-1/miR-215 gene were downloaded. Homologous analysis was used to identify and download 2 kb putative promoter sequences of rat and human pri-miR-194-1/miR-215 genes. HNF1a was found to induce the expression of miR-194 in the intestine.[17] HNF1a, HNF4a, and HNF4b were found to be expressed preferentially in the liver, intestine, and kidneys.[18], [19] The promoter sequence of pri-miR-194-2/miR-192 and pri-miR-194-1/miR-215 was then analyzed using Motif-based sequence analysis tools (http://meme.nbcr.net). A conserved HNF1 binding site and a conserved HNF4 binding site were found in the pri-miR-194-2/miR-192 gene promoter region with expected *P* values below 0.001 (**Figure 6**). However, no conserved HNF1 or HNF4 binding sites were found in the pri-miR-194-1/miR-215 gene promoter. pri-miR-194-2/miR-192 and pri-miR-194-1/miR-215 gene promoters were found to be conserved across HNF4 binding sites, but a two-nucleotide deletion, CC, was observed in the HNF4 binding motif in pri-miR-194-1/miR-215 gene promoters. This may be connected to the loss of expression of pri-miR-194-1/miR-215 genes in the kidneys and liver.

## Discussion

Strategies for distinguishing the tissue expression pattern of paralogous miRNA precursors that share the same major mature form are here discussed. The hairpin miRNA precursor is processed into a major/minor pair. Because the minor form is less conserved and more likely to differ across paralogous precursors, it is possible to distinguish those paralogous precursors by the minor form that they produce. Products of minor miRNA have been successfully used to distinguish miR-2a-1 and miR-2a-2 in Drosophila.[20] Here, the statistical significance of the occurrence of major/minor pairs in specific tissues was computed using online datasets showing mice miRNA deep sequencing information. Based on the coexpression score of the major/minor pair and Fisher exact test, 19 of 42 paralogous families were found to be differentially expressed in tissues (**Table 2** and **Table S3**). No differential expression was detected among miR-124-1, miR-124-2, and miR-124-3 or among miR-9-1, miR-9-2, and miR-9-3, which are specifically expressed in the brain, using this approach because they produce the same major and minor forms. Real-time PCR was developed to determine the expression of 70 nt hairpin miRNA precursors.[21] Direct analysis of precursor expression through real-time PCR may also be used to discriminate the paralogous family, including precursors that could not be distinguished. However, these paralogous precursors have high degrees of sequence identity. The primers designed in this way may have partially or completely identical sequences. In this way, specificity is an important factor and must be considered. In the present study, deep sequencing data was used to distinguish the expression of paralogous precursors based on differences as slight as a single nucleotide in the minor form.

Several studies have reported strand selection of miRNA expression.[10], [22], [23] About half of all miRNA duplexes have been found to have a highly dominant strand, as indicated by comparison of miRNA/miRNA* ratios across the miRNA sequence libraries.[24] Due to strand selection bias, the proportion of reads derived from each arm of a given hairpin can differ greatly across different tissues, even to the extent that the major and minor forms can switch. To assess the prevalence of changes in arm usage in the datasets, expression ratios between major and minor miRNAs were determined in 419 pairs with more than 50 reads. A broad range of differences were found. An expression ratio greater than 400 was observed for 135 miRNA pairs, but it was less than 10 for 132 miRNA pairs (see **Table S4**). miRNAs with the expression ratios around 2 were found to switch major and minor forms in different tissues. Strand selection bias exists for some miRNA pairs in these datasets. Strand selection bias can produce differences in the levels of expression within some miRNA pairs. The details of selection mechanisms have not yet been fully determined. The sequence features of mature forms are likely to be linked to strand selection bias within the human miRNA pairs.[25] Because members of miRNA precursor families are processed to the same major form, strand selection bias may be similar. Major and minor forms are processed from the same precursor, so the expression of major and minor forms may be correlated. We found that the expression of 73 percent of major and minor pairs was positively correlated by Pearson's correlation analysis. It is reasonable to distinguish the expression of multiple hairpins that express the same major mature miRNA by looking at the differences in the co-expression scores of major/minor pairs. Statistical analyses were used in the present study. Only the top 10% of the scored major and minor pairs were selected. The two tailed Fisher's exact test was performed to further justify the selection. Again other strategies (co-expression of host gene and cluster members) were used to validate the identification.

More than half of known mammalian miRNAs are located in the same transcription unit as protein-coding RNAs and other noncoding RNAs.[26] These miRNAs are transcribed in parallel with their host genes.[11] Several studies assess the correlation of expression between intragenic miRNAs and their host genes. 41 intragenic miRNA/host gene pairs were found to be positively correlated using NCI-60 data.[27] In addition, evolutionarily conserved intragenic miRNAs tend to be co-expressed with their host genes in human based on miRNA and mRNA microarray data.[28] miRNA precursors from the same paralogous miRNA family may be located in different host genes. In this way, the expression patterns of paralogous precursors can be distinguished by determining the expression of microRNA host genes. In the current study, the patterns of expression of mmu-miR-128-1 and mmu-miR-128-2 were clarified by assessing the expression of host genes R3HDM1 and ARPP21. miR-128-3p is the common major form of mmu-miR-128-1 and mmu-miR-128-2, and it was found to be selectively expressed in the brain and thymus (**Table 1**). The major/minor pair miR-128-3p/miR-128-2-5p was found to have a much higher coexpression score in the thymus than the pair miR-128-3p/miR-128-1-5p, but this was not the case in the brain (**Table 2**). The expression of ARPP21 and R3HDM1 was assessed in 16 mouse tissues. Results showed ARPP21 to be selectively expressed in the brain and the thymus, but R3HDM1 was not (**Figure 3C and 3D**). The expression of the minor mature forms miR-128-1-5p and miR-128-2-5p were investigated in 16 mouse tissues. Like the host gene ARPP21, miR-128-2-5p was preferentially expressed in the thymus and the brain (**Figure 3E and 3F**). Moreover, the expression of miR-128-3p and ARPP21 was significantly correlated by Pearson's correlation analysis. These results suggest that mmu-miR-128-1 and mmu-miR-128-2 are differentially expressed in the thymus. mmu-miR-128-2 is the major contributor to the expression of miR-128-3p in the thymus. Both mmu-miR-128-1 and mmu-miR-128-2 contribute to the expression of miR-128-3p in the brain.

miRNA genes are distributed across chromosomes either individually or in clusters. A miRNA cluster is a group of miRNA genes located within a short distance of each other on the same chromosome. The miRBase database defines this distance as 10 kb.[29] Almost half of *D. melanogaster* miRNA genes are grouped in genomic clusters.[30] Although miRNAs from almost all clusters have similar tissue expression profiles, some clusters contain miRNAs with uncoordinated expression profiles. The expression of 80 percent of clustered miRNA pairs were positively correlated in our datasets. The post-transcriptional regulation of miRNA maturation can be considered a possible cause of uncoordinated expression of clustered miRNAs.[30] Paralogous precursors that share the same major form may be derived from different miRNA clusters. The expression of these precursors can be distinguished by determining the expression of cluster miRNAs. miR-194-5p, which is the common major form of miR-194-1 and miR-194-2, was found to be selectively expressed in the kidneys and liver (**Table 1**). The miR-194-1/miR-215 and miR-194-2/miR-192 clusters were processed into the same mature form miR-194-5p. Using major/minor coexpression analysis, mmu-miR-194-2 was found to be preferentially expressed in the liver and kidneys, but not mmu-miR-194-1 (**Table 2**). The miR-194-1/miR-215 and miR-194-2/miR-192 cluster served as an example and is here used in a demonstration of how to distinguish the expression of paralogous miRNA genes by analyzing the expression of cluster members. miR-194-5p and miR-192-5p are co-expressed in the liver and kidneys. Using real-time PCR, it was confirmed that mmu-miR-194-2 is preferentially expressed in the liver, kidneys, and the gastrointestinal tract by analyzing the expression of miR-194-5p, miR-192-5p, and miR-215-5p in 16 mouse tissues (**Figure 4A, 4B, and 4C**). Because some clusters were found to contain miRNAs with uncoordinated expression profiles, it is necessary to use coordinated clusters to distinguish the expression of paralogous miRNA genes.

The mechanism underlying the differentially expression of paralogous precursors is as follows. Transcription of the promoter and the processing of pri-miRNAs and precursors can affect miRNA expression. The mechanism by which mmu-miR-194-1 and mmu-miR-194-2 are differentially expressed in tissues was determined. The pri-miR-194-2/miR-192 gene promoter was found to have conserved HNF1 and HNF4 binding sites. Like the pri-miR-194-2/miR-192 gene, HNF1 and HNF4 are transcription factors selectively expressed in the liver, kidneys, and intestines.[18], [19] These results suggest that the difference in the transcription binding site of the miRNA gene promoter may be linked to the differential expression of paralogous precursors in tissues.

In summary, multiple strategies for distinguishing paralogous miRNA precursors that share the same major mature form were demonstrated. These strategies may facilitate investigations of the mechanisms underlying the regulation of miRNA gene expression in different tissues.

## Materials and Methods

### miRNA sequence reads and heat map of miRNA expression

MiRBase is the primary online miRNA sequence and annotation database. Recently, short RNA deep-sequencing data were incorporated into the miRbase, facilitating detailed miRNA annotation. Each small RNA sequence read is mapped to the stem-loop sequence with detailed information of count number and start position per each experiment. A full report of annotations from miRBase release 19 was downloaded for 15 mouse tissues, including the name of precursor and mature miRNA, cluster information, the numbers and positions of mapped reads, and experimental information (http://www.mirbase.org). The reads that mapped to the same start position for each miRNA precursor without any mismatch under experimental conditions were then recorded. The miRNA guide strand (processed into the major form) and passenger strand (processed into the minor form) arm were defined for each miRNA precursor. The position with the highest number of reads was considered the location of authentic major mature miRNA and of guide strand arm. The highest sum of reads was used to calculate the expression of major form. The alternative arm from the same precursor was here defined as the passenger strand, and the highest number of reads in this strand was used to calculate the expression of the minor form. Each miRNA count from each type of mouse tissue was normalized to the total number of reads for that type of tissue. The normalized reads were used to build a heat map using GenePattern software.[31] To determine the prevalence of changes in arm usage in 15 mouse tissues, expression ratios between major and minor miRNAs in 419 pairs with more than 50 reads were determined. miRNA deep sequencing data for human colon cell lines were downloaded from the GEO database in NCBI under accession numbers GSM416758 and GSM416759. Mapped reads that showed no mismatches for miR-194-5p, miR-192-5p, and miR-215-5p were extracted.

### Tissue coexpression of major/minor miRNA pairs

Tissue coexpression scores of major and minor miRNAs were determined using previously described methods.[32], [33] Mapped microRNA reads are summarized in one matrix. The matrix showed one column per tissue and one row per miRNA, including precursors, major forms, and minor forms. miRNAs with total reads more than 50 were selected. There were 237 miRNAs in 15 different tissues. These miRNAs were classified into 42 paralogous families.

For an array, define the following:

Mij: sequence reads for miRNA i in tissue j

Ti: total reads of miRNA i in all 15 tissues

Qj: the ratio of the count of all miRNAs in tissue j to the total count of sequence reads of all miRNAs in all 15 tissues.

Zij: the tissue specificity of miRNA i in tissue j
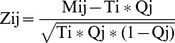



Then the tissue coexpression score of major and minor form of miRNA i in tissue j was defined as follows:
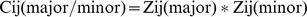



The top 10% of the scored major and minor pairs were selected, and then a two-tailed Fisher's exact test was performed to assess the differential expression of minor forms that are processed from the same family of precursors. The minor forms with *P* values below 0.05 were used as statistical justification for the further selection of major and minor pairs to discriminate the expression of miRNA precursor.

### Pearson's correlation coefficient and statistical significance

Pearson's correlation coefficient (R) is a measure of the strength of the correlation between the two variables. It is widely used as a measure of gene coexpression. To test the association between paired major and minor miRNAs, among clustered microRNAs, and between intragenic miRNAs and host genes, the R-value and p-values were computed.[34]–[36]

### PaGeFinder analysis of tissue-specific expression of mature miRNA

Pattern Gene Finder (PaGeFinder) is a free online server to analyze the tissue expression pattern of genes.[37] PaGeFinder implements new algorithms and functions for quantitative identification of pattern genes like specific and selective genes, housekeeping genes, and repressed genes. Tissue-specific expression of mature miRNAs was performed using PaGeFinder analysis. The normalized reads of major mature miRNAs were subjected to analysis. The search criteria was set at SPM>0.9 for the identification of miRNAs that underwent tissue-specific expression or at SPM>0.5 and CTM>0.9 for the identification those that underwent tissue-selective expression.

### Isolation of RNA from mouse tissues

C57BL/6J mice aged 8–12 weeks were purchased from the Soochow University Animal Center. They were housed in a temperature- and humidity-controlled room and supplied with rodent chow and water. Mice were anesthetized intraperitoneally with ketamine and xylazine and then killed. Samples of the thymus, spleen, brain, eye, lung, heart, liver, kidney, adrenal gland, urinary bladder, stomach, intestine, skin, skeletal muscle, testis, and ovary were collected. The animal experiment in this study was approved by the Soochow University Animal Center, Jiangsu Province, China. Total RNA was extracted and isolated by using Tri Reagent from about 30–50 mg mouse tissues according to the manufacturer's instructions. RNA quality and quantity were assessed using agarose gel electrophoresis and A260/A280 ratio and A260/A230 ratio with spectrophotometer. The A260/A280 ratios for all RNA preparations were greater than 1.9, and the A260/A230 ratios for all RNA preparations were greater than 2.0.

### miRNA quantitative real time PCR

Quantitative real-time PCR (qRT-PCR) was used to determine the expression of mature miRNA in different tissues in mice, as previously reported. The primers are listed in **Table S1**. In brief, total RNA were treated with DNase and purified using phenol/chloroform extraction and ethanol precipitation. Then 1 µg of the DNase-treated RNA was poly A tailed and purified using phenol/chloroform extraction and ethanol precipitation. These poly-A-tailed RNA were reverse-transcribed into cDNA with polyT adapter as the primer. The real-time PCR thermal conditions for all mature miRNAs were 95°C for 10 min, followed by 40 cycles of 95°C for 15 s and 60°C for 60 s. Data were analyzed using relative real-time PCR quantification based on the comparative CT method. The endogenous reference gene was U6 snRNA. Single-factor ANOVA (*P*<0.05) followed by Turkey's honestly significant difference (HSD) test was used to examine the tissue types in which a miRNA expressed preferentially.[38]_ENREF_27

### mRNA quantitative real time PCR

Quantitative real-time PCR (qRT-PCR) was used to determine the expression of host gene in different tissues in mice. The primers are listed in **Table S1**. In brief, 1 µg of total RNA was reverse-transcribed into cDNA with dT17, random hexamer primer and MMLV reverse transcriptase. The real-time PCR thermal conditions for all the selected genes were 95°C 10 min, followed by 40 cycles of 95°C for 15 s and 60°C for 60 s. The expression of genes relative to 18S RNA was determined using the comparative CT method. Single-factor ANOVA (*P*<0.05) followed by Turkey's honestly significant difference (HSD) tests was used to determine the type of tissue in which each gene was preferentially expressed.

### Analysis of miRNA promoter transcription

Promoter sequences of the pri-miR-194-2/miR-192 and pri-miR-194-1/miR-215 genes were downloaded from the UCSC website. Transcription binding sites were predicted using motif-based sequence analysis tools (http://meme.nbcr.net). Promoter DNA sequences were aligned using Vector NT software.

## Supporting Information

Table S1 Primers.
(XLSX)

Table S2 miRNA reads.
(XLSX)

Table S3 Coexpression score of major/minor pairs.
(XLSX)

Table S4 Expression ratios between major and minor forms.
(XLSX)

Table S5 Pearson correlation analyses between major and minor pairs.
(XLSX)

Table S6 Pearson correlation analyses among cluster miRNAs.
(XLSX)
